# The occurrence of pristine and functionalized fullerenes as constituents of airborne aerosols

**DOI:** 10.1038/s41598-023-31119-4

**Published:** 2023-03-14

**Authors:** Fábio N. dos Santos, Madson M. Nascimento, Gisele O. da Rocha, Jailson B. de Andrade

**Affiliations:** 1 Centro Interdisciplinar de Energia e Ambiente-CIEnAm, Universidade Federal da Bahia, Salvador, BA 40170-115 Brazil; 2 ThoMSon Mass Spectrometry Laboratory, Institute of Chemistry, University of Campinas, Campinas, São Paulo 13083-970 Brazil; 3 Institute of Chemistry, Universidade Federal da Bahia, Salvador, BA 40170-290 Brazil; 4 Centro Universitário SENAI-CIMATEC, Av. Orlando Gomes, 1845-Piatã, Salvador, BA 41650-010 Brazil; 5 Instituto Nacional de Ciência e Tecnologia em Energia e Ambiente-INCT, Universidade Federal da Bahia, Salvador, BA 40170-115 Brazil

**Keywords:** Environmental sciences, Chemistry

## Abstract

We investigated if pristine and functionalized fullerenes could be actual constituents of fine atmospheric aerosols. Comprehensive profiles of fullerenes from 1 µL extracts were made through matrix laser desorption ionization Time-of-Flight Mass Spectrometry (MALDI-MS) within a few minutes. The ion with m/z 720, corresponding to [C_60_]^−•^, was identified as fullerene after 1 µL of α-cyano-4-hydroxycinnamic acid matrix solution was spotted over the dried extracts. The ions with the m/z corresponding to C_70_, C_76_, C_84_, C_100_, C_118_, C_128_, and C_130_ were also attributed to other fullerene species detected within the samples. The ion m/z 878 was found to be the fullerene derivative diethyl methano[60]fullerene dicarboxylate. Since ions of fragmented fullerene molecules were not detected even at high laser energies, we considered the fullerenes’ occurring as original constituents of real atmospheric particle matrices instead of being formed as artifacts of the laser action on samples. Therefore, this protocol would be helpful in the understanding of the distribution of either pristine or functionalized fullerenes in the environment and their participation in atmospheric chemistry under typical conditions, as well as its application in vitro and in vivo (eco)toxicity studies.

## Introduction

Pristine or unsubstituted fullerenes (such as C_60_ and C_70_, among others) and their derivatives (functionalized fullerenes) are compounds formed mainly by carbon atoms arranged in a spherical shape as buckyballs^1–5^. The chemical versatility of fullerenes and their derivatives makes them useful for an extensive array of applications, including energy generation^6^ electronics, optics, photovoltaics, in (bio)medicine, and personal care products, among others^7–9^. Indeed, until 2012, an estimate of the production of fullerenes was approximately tens of thousands of tons per year^7,10^. Even though from 2014 to 2019, the production of fullerenes increased by about 6% only^11^, their production will probably increase shortly.

The actual occurrence of fullerenes in the environment still is not a consensus in the literature^3,12^, despite the fact they have been found in sewage and surface waters^13^, sediments^14–16^, soils^7,14^, engine soot^17^, airborne particles exhausted from coal^18,19^, and diesel^20^ burnings, and meteorites^2^. The presence of fullerenes in the environment has been proposed either as an actual occurrence or attributed to their formation of laser artifacts during laser desorption ionization (LDI) or matrix-assisted laser desorption ionization (MALDI) mass spectrometry (MS) analyses^12^.

Fullerenes and derivatives have been related in a few studies, probably because their occurrence in environmental or biological samples is hard to detect^4,12^. Another reason is that fullerenes may suffer atmospheric transformation under several processes, including aggregation, coating, and reactions such as oxidation^21^ and photooxidation^22^. Some studies have conducted an exploratory search on the chemical composition of metals and the water-soluble organic fraction of fine aerosols (PM2.5), finding mainly metals originating from industrial (Cu, Cd, and Pb) and traffic (Cr, Mn, Ni, V, and Zn) emissions as well as those from natural emissions (Na, K, Ca, Ti, Al, Mg, and Fe)^23^. However, it has not been related to any influence of the metal content on forming the fullerene derivatives. Fullerenes generally occur at trace or ultra-trace levels, which make them challenging to quantitatively extract under conventional methods. Accordingly, they may also be hard to detect and be quantified in environmental matrices when considering hundreds to thousands of other sample components, which may act as interferents, depending on which sample extraction and analysis technique is employed.

Currently, there is still a lack of methodologies for comprehensive extraction, detection, and identification of several (pristine and functionalized) fullerene compounds in complex matrices. Recently published studies have primarily focused on the C_60_ and C_70_, as well as a few substituted fullerenes^4^. Analytical approaches to comprehensive detection and quantification are challenging, especially in more complex biological (e.g., urine, blood, plasma, milk, and tissue) and environmental (soil, water, sediments, and atmospheric aerosols) matrices. In addition, this limits studies regarding exposure-risk assessment and the determination of possible biological effects related to the fullerenes^24^.

Fullerenes have been extracted from environmental samples such as water^25^, soil^7,14^, and sediments^14^, most commonly through liquid–liquid extraction (LLE) using toluene or, more recently, through ultrasound-assisted dispersive liquid–liquid microextraction^23^. Soxhlet extraction, sonication, and supercritical protocols have also been tested in relation to their capacity to extract fullerenes from carbonaceous materials. However, yields well below 5% for the C_60_ extraction were obtained for all the carbonaceous matrices.

Regarding the analysis step, different types of ionization sources, such as APPI, ESI, and APCI, have been used, but only a few studies have applied MALDI-MS. Different mass spectrometry platforms, such as chromatography-free setups have been used to characterize fullerenes, with different ionization sources such as electrospray ionization (ESI), atmospheric pressure chemical ionization (APCI), atmospheric pressure photoionization (APPI), laser desorption ionization (LDI)^12^ and matrix assisted laser desorption ionization (MALDI)^12^, and different mass analyzers (such as Time-of-Flight (TOF) and Ion Mobility)^3^. LC–QTOF–MS was also applied to determine the pristine fullerenes C_60_ and C_70_, in addition to six methanofullerenes, such as the [6,6]-phenyl-C61-butyric acid methyl ester ([60]PCBM) in soil samples^7^.

Liquid chromatography-mass spectrometry (LC–MS) has also been used for fullerene quantification^23^. For instance, Carboni et al.^14^ developed an analytical method for soil and sediment matrices that reached instrumental method limits of detection (ILoD) at 6 and 12 fg for C_60_ and C_70_, respectively. In the same study, the instrumental method limits of quantification (ILoQ) were 20 and 39 fg, respectively, for C_60_ and C_70_^14^. On the other hand, quantification by MALDI-MS is often recognized as a limited technique due to its lower reproducibility and the highest relative standard deviation. However, some studies have presented significant progress, presenting MALDI-MS results comparable to those from LC–MS quantifying nitroaromatic compounds in contaminated soils^24^.

Nonetheless, there is a need for robust, sensitive, and comprehensive analytical methods for fullerenes to better understand possible fullerene-related adverse effects in human beings and animals. In fact, toxicity and ecotoxicity studies are still limited nowadays due to the lack of a methodology for the determination of pristine and derivatized fullerenes that would be able to unequivocally detect them as actual constituents of environmental samples^14^. The same applies to atmospheric samples.

Since fullerenes may work as intra-matrices (matrices themselves), they would be detected in the atmospheric particulate matter as actual constituents. In the present study, we used fine atmospheric particulate matter samples (PM2.5). These samples were used to determine if fullerenes are among their original constituents. Then, we performed a comprehensive profile of fullerenes using the matrix-assisted laser desorption ionization (MALDI) technique in a chromatography-free setting. The results are critically discussed.

## Results

### Optimization of the MALDI matrix and signal of analytes

**Figure 1:**
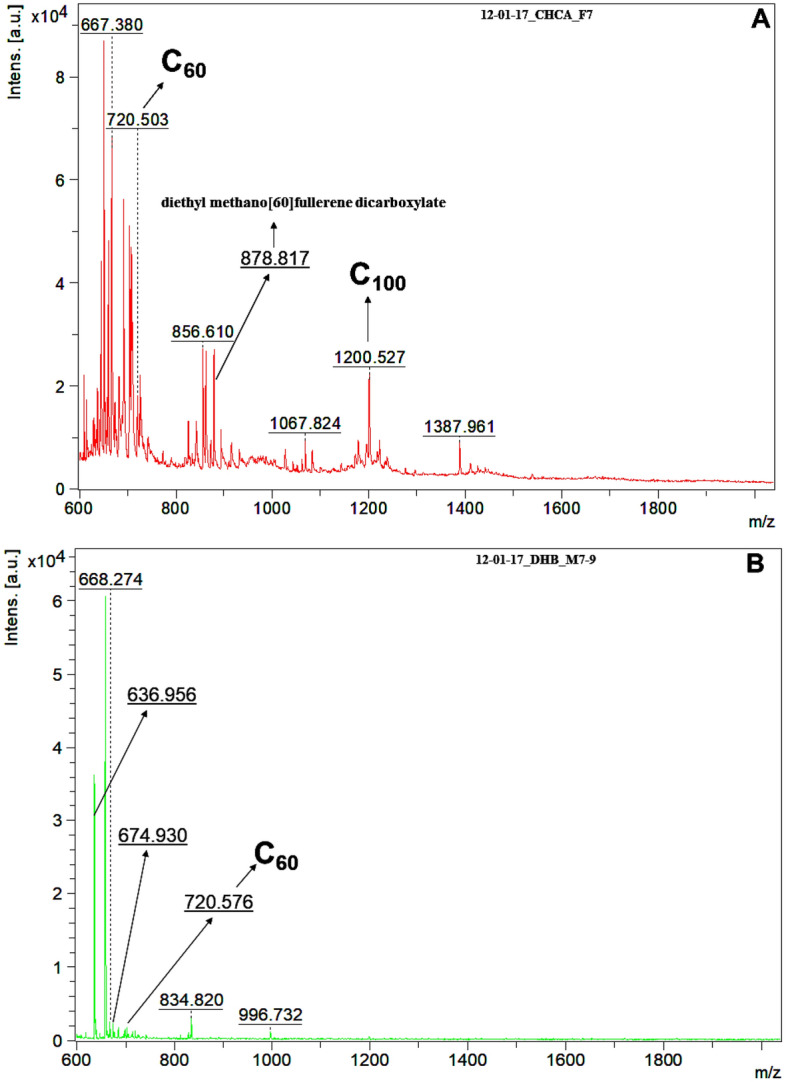
MALDI-MS profile of a fine aerosol sample. Typical spectra of the filter samples showing some fullerenes and matrix effect of the α-cyano-4-hydroxycinnamic acid—CHCA (**A**) and the 2,5-dihydroxybenzoic acid—DHB (**B**) matrices on detected fullerene ions.

To begin, matrices for fullerene analyses were tested using 2,5-dihydroxybenzoic acid (DHB) (commonly used in hydrocarbon analyses) and α-cyano-4-hydroxycinnamic acid (CHCA) (commonly used in peptide or protein analyses). For this essay, a sampled filter containing PM2.5 particles collected on Dec-01-2017 was used. The CHCA presented a better MALDI matrix to fullerene than DHB over the mass range of interest (600–2000 Da). It can be noted by the number of detected ions and their abundance when spectra from Fig. 1 are compared to each other. For instance, the ion with m/z 720 (C_60_) was more abundant using the CHCA instead of the DHB matrix, and the detection of more species was also improved when using the CHCA.

**Figure 2:**
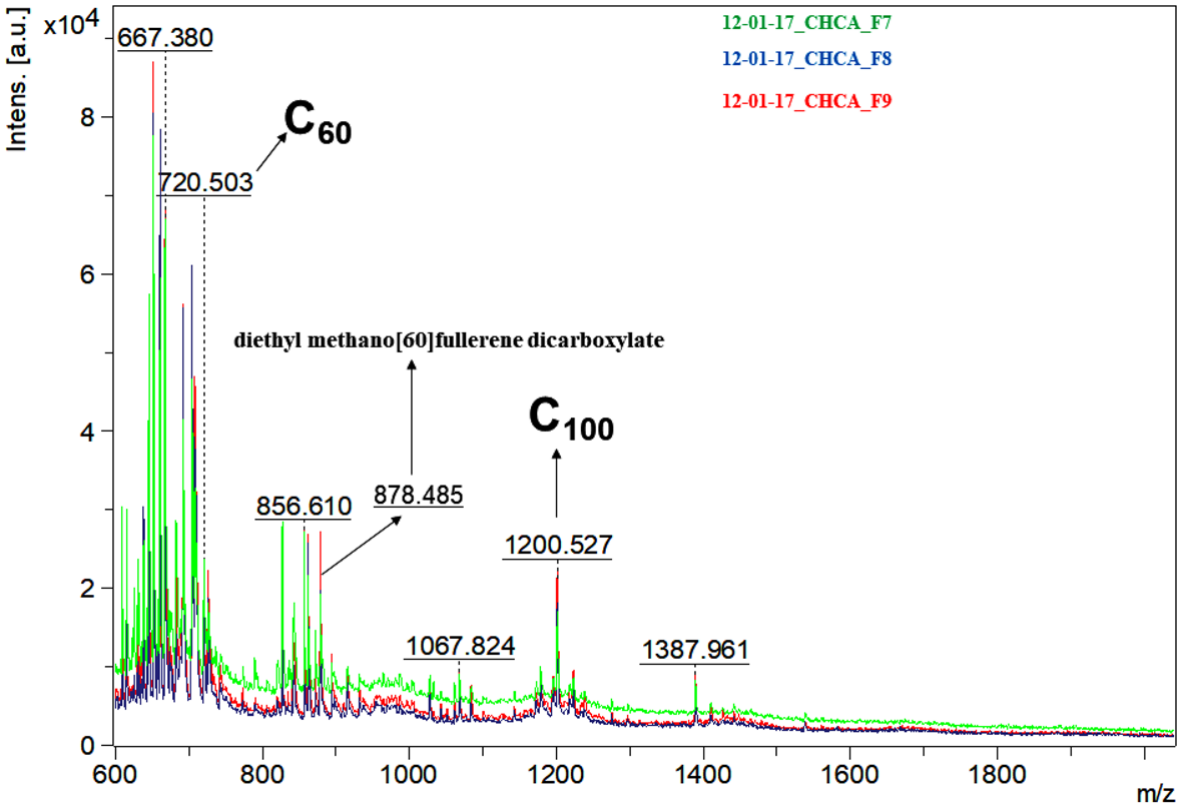
MALDI-MS profile of one fine aerosol sample analyzed in triplicate. Typical spectra of the sample presenting high repeatability between three replicates using the CHCA matrix.

After choosing CHCA as the MALDI matrix for this study, we investigated if the signals and profiles of three portions of the same filter sample (sampled on Dec-01-2017) were extracted, under successive analyses, would be similar. Indeed, the obtained spectra presented high repeatability to three true replicates (Fig. 2). Accordingly, the blank filter spectra are presented in Supplementary Data (Figs. S1, S2, and S3).

Next, we applied matrix-assisted laser desorption ionization mass spectrometry (MALDI-MS) analyses to detect fullerenes in real atmospheric sampled filters. During analysis, samples underwent photoionization and did not require any additional cationizing salts.

**Figure 3:**
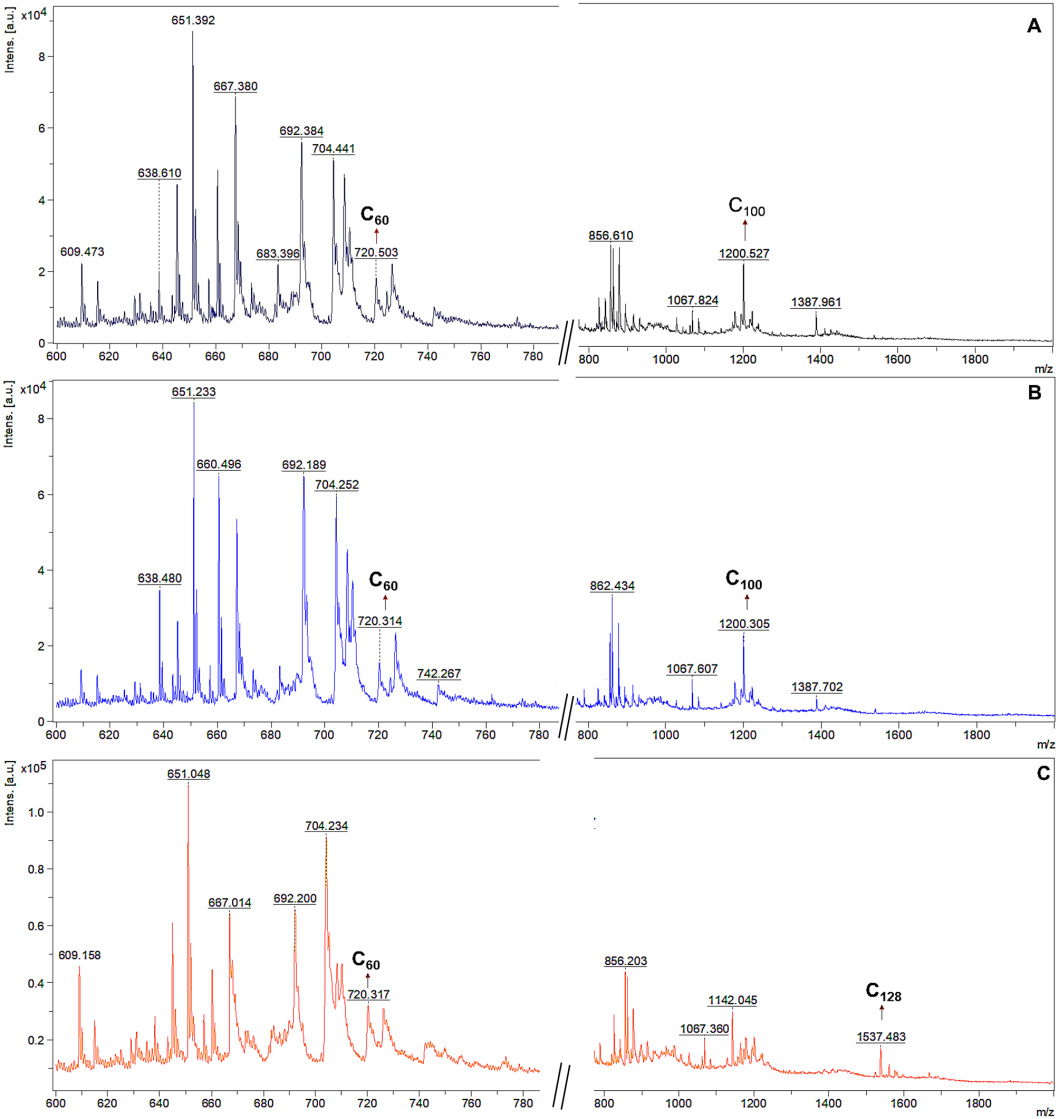
MALDI-MS spectra from fine particle samples collected at Dec-1st-17 (**A**), Dec-13th-17 (**B**) and Dec-14th-17 (**C**) (from the top to down). Those sample spectra presented the fullerene core of the molecule observed as the ion with m/z 720 (C60) using the CHCA matrix.

**Figure 4:**
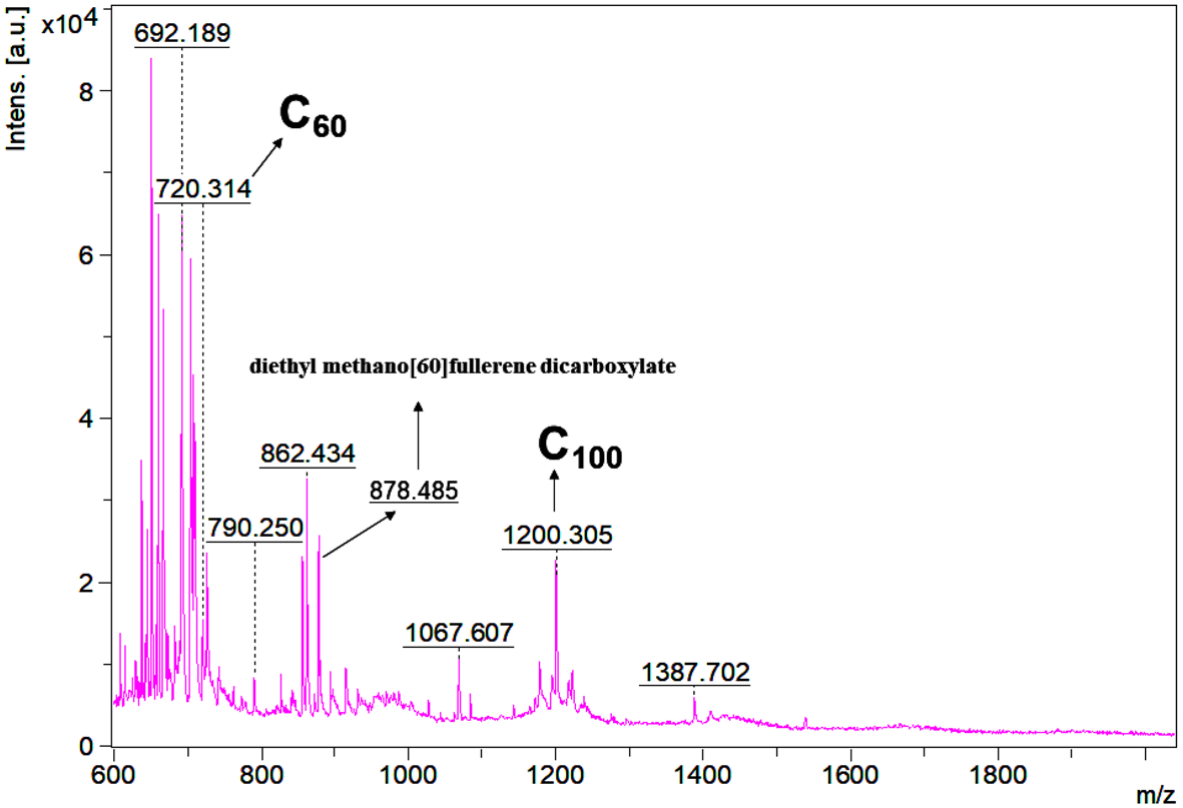
MALDI-MS spectra from a fine particle sample showing the fullerene diethyl methano[60]fullerene dicarboxylate observed as the ion with m/z 878.

The fullerene ion with m/z 720 (C_60_) was detected with high mass accuracy in different samples (Fig. 3), as well as the diethyl methano[60]fullerene dicarboxylate derivative (Fig. 4). It is noteworthy to mention that it was not found any indication in the literature of this derivative in atmospheric samples. To the best of our knowledge, this may be the first time this fullerene derivative is found in this kind of sample. Because the fullerene compounds in the current study are genuine atmospheric aerosol constituents, we hypothesize that diethyl methano[60]fullerene dicarboxylate was formed during particle aging through long-range transportation*.* It may have been formed by either photooxidation by OH radicals, ozonolysis, oxidation, photolysis, or reaction with NO_3_ radicals of the C_60_ fullerene. Since the region we collected the samples is remote, we believed the derivative precursor (the C_60_ fullerene) may have originated far from the sampling points and then suffered a number of modifications all the way until we collected it.

## Discussion

### Extraction procedure

In this study, we employed a miniaturized sample preparation procedure to extract fullerenes combined with MALDI-MS for the first time, by employing some of the principles of Green Chemistry. Some of the aspects of Green Chemistry considered here are: (i) minimization of the sample amount; (ii) reduction of reagents and energy consumption; (iii) miniaturization and automation; (iv) reduction of waste; (v) multi-analyte determination; (vi) improvement of analyst safety; and (vii) the replacement of toxic reagents by less toxic alternatives^26^. Both the extraction and MALDI analysis procedures have been shown to be decisive tests to approach a comprehensive analysis of fullerenes. In our previous studies, several polycyclic aromatic compounds were successfully quantified in fine aerosols using miniaturized extraction devices^27–29^. However, using a GC–MS with an electron ionization source and a typical low-resolution mass spectrometer analyzer were the major limiting factors to detecting more complex PACs or fullerenes at that time. Our miniaturized and low-consuming solvent extraction procedure has shown a low sample amount needed for analysis (only 2% of the total filter area, 4.15 cm^2^). This reduction also reflects the employment of organic solvent volumes as low as 500 μL. In the present work, we replaced more toxic and less environmentally friendly solvents (such as dichloromethane^30^, which has been discouraged since the Montreal Protocol) with ethyl acetate, which has many analytical advantages. Therefore, in terms of the prerogatives of Green Chemistry, our extraction method is more advantageous.

### Fullerenes in atmospheric particles: original sample components *vs.* artifact formation

Here, the primary hypothesis to be investigated is whether fullerenes would potentially be original constituents of aerosol samples. And if it is the case, they actually occur in the real fine air particles as pristine or substituted fullerenes to be detected in those samples. In fact, there were recently reported some pieces of evidence of fullerenes from particles exhausted from coal combustion^19^ and diesel engine soot^20^. Indeed, Wang et al.^18^ pointed out that fullerenes may be part of the black carbon (BC) or brown carbon (BrC) portion of atmospheric particles collected in ambient air in Eastern China. Schnaiter et al.,^31^ on the other hand, suggested fullerenes would be components of the aerosol particles in fresh snow samples collected from Germany.

On the other hand, some other studies have found that fullerenes could be potentially produced as artifacts due to the laser energy during the ionization process. This is especially true when the matrix-free protocol Laser Desorption Ionization (LDI)^12^ is used for the analysis certain types of geological samples. Therefore, if fullerenes had artifact-related occurrences, there would probably be a fullerene abundance-dependence directly proportional to the applied laser energy. If that were the case, mass fragments shorter than m/z 720 (from the C_60_) would be frequently detected within the sample spectra. However, there is not enough evidence in the literature about the fullerenes’ abundance-dependence from applying different laser energies. There is neither evidence of extensive fragmentation associated with high laser energy nor enough comparison of the Laser Desorption Ionization process in both the presence and absence of matrix.

Nonetheless, some studies have demonstrated that the polycondensed aromatic and fullerene molecules may work as “self-matrices”^32^. Based on this evidence, fullerenes would be detected in atmospheric particles independently of the presence of a matrix (LDI or MALDI). Since those studies have detected fullerenes by the LDI technique^12^, we investigated if the matrix type would also influence the fullerenes’ detection. Our results showed that different types of matrices could have affected whether the fullerene ion intensities went up or down when laser energy was used. This is because laser energy can help desorption/ionization processes happen in addition to ionic suppression. We simultaneously detected many fullerene compounds through the matrix laser desorption ionization analysis. The ion at m/z 720, corresponding to [C_60_]^•+^ was annotated as a fullerene compound (Fig. 3). Additionally, the ion with m/z 840 is related to the [C_70_]^•+^ species. The species C_76_, C_84_, and C_100_, attributed to fullerene derivatives, were also detected in the samples (Fig. 3) (Table 1). Given that: (i) MALDI source generally is recognized as a smoother desorption/ionization method (in comparison to other ionization methods), and (ii) our results did not present any detectable fragmented fullerene ions even under high laser energies, we believe fullerenes are probably actual constituents of fine aerosols.

**Table 1:** Detected fullerenes and their derivatives detected in the MALDI-TOF analyses using CHCA as matrix.

Compound	PM2.5 Sample (date of sampling)	Experimental m/z	Fraction of fullerene^a^ (%)	Species
C_60_	Dec-01–2017	720.3	1.86	[M]^•+^
C_70_	Nov-11–2017	840.6	0.71	[M]^•+^
C_74_	Dec-13–2017	888.3	0.76	[M]^•+^
C_76_	Nov-19–2017	912.5	1.89	[M]^•+^
C_84_	Dec-14–2017	1008.4	1.25	[M]^•+^
C_92_	Dec-24–2017	1104.5	0.80	[M]^•+^
Fullerene diethyl methano[60]fullerene dicarboxylate	Dec-01–2017	878.2	1.78	
C_100_	Nov-06–2017	1200.5	4.68	[M]^•+^
C_118_	Dec-24–2017	1416.4	0.97	[M]^•+^
C_128_	Nov-06–2017	1537.2	4.65	[M]^•+^
C_130_	Nov-19–2017	1560.4	2.24	[M+H]^+^

^a^Calculated from fullerenes peak areas.

### MALDI matrix effect based on evidence of the original occurrence of fullerenes in fine aerosols

We also observed a strong matrix effect in the profiles of the fullerenes based on their signal intensities and the number of ions detected. Those findings point out the importance of studying and optimizing the type of matrix to be used in order to enhance the MALDI-MS analyses (Fig. 1). The CHCA matrix, commonly used in the MALDI-TOF for analyzing peptides and proteins, presented twice as many ions with higher intensities than those from the DHB matrix (Fig. 1).

High miscibility between the MALDI matrix solution and the sample solution during the spot preparation step is crucial for improving the ionization of the analytes and then the desorption of the ions into the gas phase during the laser impact. It could also minimize the unwanted fragmentation of the analytes’ molecules during the MALDI analysis. Furthermore, the intermolecular interactions among the fullerenes’ aromatic cores and the matrix during the photoionization step and the intermolecular charge transferences result in a decrease in the probability of the ionization of the analytes^32^. Our results have demonstrated that the fullerenes’ desorption/photoionization processes in atmospheric particles are more favorable when using the CHCA matrix because more fullerene ions at their highest intensities were detected^32^.

Fullerenes or derivatives quantification by MALDI-MS remains difficult because the matrix/analyte ratio has a strong influence on the correlation between signal intensity and analyte amount. The ion suppression effect has also been reported due to strong intermolecular interactions among the hexabenzocoronene (HBC) aromatic cores^32^. However, few studies have been reported dealing with the quantitative MALDI-MS analysis of the C_60_ and C_70_ fullerenes because the spherical C_60_ and C_70_ buckyball structures prevent them from having strong aggregation with the matrix ions. While the ionic suppression effect on the fullerene quantification analysis is insufficient, no molecule-dependent anomalies in their desorption-photoionization behavior are recognized^32^.

## Conclusion

In conclusion, our study presented strong evidence that pristine and substituted fullerene compounds are actual constituents of fine atmospheric aerosols. Based on the number of detected species and their relative ion intensities, we demonstrated that α-cyano-4-hydroxycinnamic acid is suitable for detecting fullerenes. The analyses of real fine atmospheric particle samples revealed comprehensive profiles of fullerenes as well as the fullerene derivate diethyl methano[60]fullerene dicarboxylate C_60_, C_70_, C_76_, C_84_, C_100_, C_118_, C_128_, and C_130_. Our method could also be applied to potentially detect oxygenated fullerene species, such as C_60_O, C_60_O_2_, and C_60_O_3_, among others, which may be formed under typical atmospheric conditions by ozonolysis or photooxidation reactions. However, in our samples, those oxygenated derivatives were not detected.

## Methods

### Reagents and standards

Solvents used to prepare the samples were acetonitrile hypergrade for LC–MS LiChrosolv® purity ≥ 99.9% (Sigma-Aldrich, St. Louis, MO), ethyl acetate 99.7% (Sigma-Aldrich, St. Louis, USA), methanol hypergrade for LC–MS LiChrosolv® purity ≥ 99.9% (Sigma-Aldrich, St. Louis, MO). Trifluoroacetic acid was LC–MS grade, and LiChropur was ≥ 99.0% (GC) (Sigma-Aldrich, St. Louis, MO). The ultrapure water used to prepare extracts was purified by the Direct-Q water system (Millipore, Bedford, MA). MALDI matrices were α-cyano-4-hydroxycinnamic acid (CHCA) MALDI-MS grade, purity ≥ 99.0% (HPLC) (Sigma-Aldrich, St. Louis, MO) and 2,5-dihydroxybenzoic acid (DHB) MALDI-MS grade, purity ≥ 99.0% (HPLC) (Sigma-Aldrich, St. Louis, MO). The phospholipid standards used for TOF calibration were 1,2-dioleoyl-sn-glycero-3-phosphatidylethanolamine (ammonium salt) purity ≥ 99% (DOPE), 1,2-dimyristoyl-sn-glycero-3-phospho-rac-(1-glycerol) sodium salt, purity ≥ 99% (DMPG),1,2-dipalmitoyl-sn-glycero-3-phosphate monosodium salt, powder, purity ≥ 99% (DPPA), 1,2-dipalmitoyl-sn-glycero-3-phospho-L-serine sodium salt, powder, purity ≥ 99% (DPPS) obtained from Avanti Polar Lipids, Inc. (Alabaster, Al, USA).

### Atmospheric sampling

During the PIRATA expedition (November–December 2017), fine atmospheric aerosol samples (PM2.5) were collected daily on board the Vital de Oliveira hydroceanographic research ship (from the Brazilian Navy). The vessel ranged from latitudes of 0° 50′ 27.54″ N to 15° 00′ 00″ N within the same longitude of 38° 19′ 0.00.36″ W, then to 4° 39′ 57.12″ S and 35° 36′ 46.62″ W at the end of the sampling period. The atmospheric pressure was approximately 1 atm during all sampling campaign. The relative humidity, wind direction and wind velocity (regarding the ship) ranged from 73 to 76%, 69° to 197°, and 16 to 18 knots. Sampling was done using a high-volume sampler (Hi-Vol, Energética, Brazil) equipped with an inlet for classifying particles with dp < 2.5 µm, which operated at 1.112 m^3^ min^−1^ of sampling rate for 24 h. This corresponded to 1457 m^3^ of total sampled air volume. The samples were collected onto quartz microfiber filters (QFF, 203 mm × 254 mm, CAT No. 1851-865, Whatman, USA). For the weighting step, we used a Shimadzu AUY220 analytical balance (0.0001 g ± 0.1 mg). The mass of PM2.5 retained on the QFF filter after sampling ranged from 0.0100 to 0.0215 g. Five blank filters (QQF not used to collect particles) were carried to the vessel and kept within the same conditions as the actual filter samples.

### Sample preparation and MALDI-MS analysis

We performed the extraction of the fullerene compounds from fine particles (PM 2.5) employing a miniaturized extraction system composed of a glass chamber syringeless microextraction device (Whatman Mini Uniprep G2 Filters, Maidstone, UK)^27,28^. The procedure was performed using a piece of 4.15 cm^2^ from the sampled filter. This piece of filter was cut into smaller particles and transferred into the glass chamber of the microextraction device. Then, we added 500 μL of the extracting solvent mix (7:3, acetonitrile: ethyl acetate) onto the sample pieces and closed them with the micro-extractor plunger. The whole micro-extractor system was sonicated for 17 min using a VWR Ultrasonic Cleaner (Radnor, Pennsylvania, USA) at 60 kHz and 39 °C. After that, the plunger was carefully pressed down into the glass chamber filtering the extract^25^. The filter blanks were extracted in the same way the samples were.

After that, the extract was spotted (1 µL droplet) onto a MALDI plate (Bruker-Daltonik GmbH, Bremen, Germany) and then air-dried. Then, 1 µL of α-cyano-4-hydroxycinnamic acid (CHCA) matrix solution was spotted over the dried sample. A phospholipid mix standard solution composed of DOPE, DMPG, DPPA, and DPPS was also deposited on the calibration spot of the plate for performing external calibration. The CHCA matrix solution was prepared at 50 mg mL^−1^ in 7:3 ACN : water with 0.1% TFA. Accordingly, the 2,5-dihydroxybenzoic acid (DHB) matrix was prepared at 10 mg mL^−1^ in 8:2 ACN : Water with 0.1% TFA.

### MALDI-MS data analysis

MALDI-MS data acquisition was performed on a Bruker Autoflex III MALDI–TOF/TOF mass spectrometer equipped with a 334 nm smart beam laser. Profiles of fullerenes were obtained using a time-of-flight (TOF) analyzer set in reflector mode and positive ion mode with a delayed extraction of 260 ns at 20 kV of accelerating voltage. Each spectrum was manually collected as an average of 5000 laser shots (1000 laser shots at five different spot positions) applying laser energy just above the threshold. A m/z range 600–2000 was used to obtain triplicate spectra using the AutoExecute of Flexcontrol acquisition software (Version 2.4; Bruker-Daltonik GmbH). Analysis of MALDI data was done on the raw spectra in FlexAnalysis software (Bruker-Daltonik) after baseline subtraction for background removal, alignment of the spectra scale, ion selection with an S/N ratio greater than 3, and normalization of intensities.

## Supplementary Information

Supplementary Information.

## Data Availability

All data generated or analyzed during this study are included in this published article [and its supplementary information files].
