# Supplementary material for: The occurrence of pristine and functionalized fullerenes as constituents of airborne aerosols
Source: Sci Rep. 2023 Mar 14;13:4248. doi: 10.1038/s41598-023-31119-4 (PMC10015080; doi:10.1038/s41598-023-31119-4)
Supplement: Supplementary file 1 — Supplementary Information. [file 41598_2023_31119_MOESM1_ESM.doc]

# The occurrence of pristine and functionalized fullerenes as constituents of airborne aerosols

# Fábio N. dos Santos^1,2+^, Madson M. Nascimento^1,4,5+^, Gisele O. da Rocha^1,3,4,5+^, Jailson B. de Andrade^1,3,4,5+^*

_1_Centro Interdisciplinar de Energia e Ambiente - CIEnAm, Universidade Federal da Bahia, 40170-115 Salvador, BA, Brazil..

^2^ThoMSon Mass Spectrometry Laboratory, University of Campinas, Institute of Chemistry, Campinas, São Paulo, 13083-970, Brazil..

^3^Institute of Chemistry. Universidade Federal da Bahia, 40170-290, Salvador, BA, Brazil..

^4^Centro Universitário SENAI-CIMATEC, Av. Orlando Gomes, 1845 - Piatã, 41650-010 Salvador, BA, Brazilde Química, Universidade Federal da Bahia, Campus de Ondina, 40170-115, Salvador-BA, Brazil

^5^Instituto Nacional de Ciência e Tecnologia em Energia e Ambiente - INCT, Universidade Federal da Bahia, 40170-115 Salvador, BA, Brazil.

* Corresponding author: jailsondeandrade@gmail.com

+these authors contributed equally to this work

**SUPPLEMENTARY MATERIAL**

Figure S1. MALDI-MS profile of blank filters using α-cyano-4-hydroxycinnamic acid (CHCA) matrix.

Figure S2. MALDI-MS profile of blank filters using 2,5-dihydroxybenzoic acid (DHB) matrix.

Figure S3. MALDI-MS profile of fine aerosol samples. Typical spectra of the sample collected at Dec-12th-2017 presenting high repeatability between three replicates using the CHCA matrix.

Figure S1

Figure S2


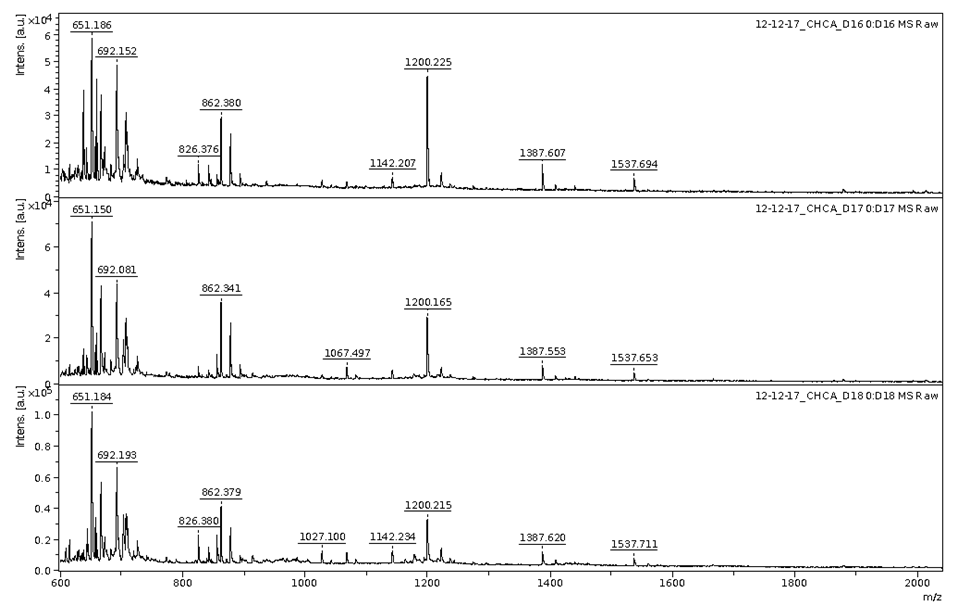


Figure S3
